# Ubiquitin-proteasome system: a potential participant and therapeutic target in antiphospholipid syndrome

**DOI:** 10.3389/fimmu.2025.1523799

**Published:** 2025-02-18

**Authors:** He Wang, Yuan Tan, Qi Liu, Shuo Yang, Liyan Cui

**Affiliations:** ^1^ Department of Laboratory Medicine, Peking University Third Hospital, Beijing, China; ^2^ Core Unit of National Clinical Research Center for Laboratory Medicine, Peking University Third Hospital, Beijing, China; ^3^ Institute of Medical Technology, Peking University Health Science Center, Beijing, China

**Keywords:** APS, UPS, E3 ubiquitin ligases, proteasome inhibitor, PROTACs

## Abstract

APS (antiphospholipid syndrome) is an autoimmune disease characterized by thrombosis, pregnancy complications and persistent elevation of aPLs (antiphospholipid antibodies). Dysfunction of innate immune cells, ECs (endothelial cells), platelets and trophoblast cells are central to the development of APS. The UPS (ubiquitin-proteasome system) is a highly conserved post-translational modification in eukaryotes. Imbalance of the UPS potentially disrupts the protein homeostasis network and provokes prothrombotic and proinflammatory signaling during APS progression. *In vivo*, low-dose proteasome inhibitors are believed to effectively inhibit the production of proinflammatory factors and the clinical manifestations of APS. In this review, we would like to summarize the likely contribution of dysregulated UPS to the pathogenesis of APS. Given the significant progress made in understanding the molecular mechanisms of the UPS and how alterations in the UPS lead to the development of autoimmune diseases, targeting the UPS may represent a novel therapeutic strategy.

## 1 Introduction

APS is a heterogeneous autoimmune disorder characterized by arterial, venous, or small vessel thrombosis and pregnancy morbidity, all associated with the presence of autoantibodies, including aCL (anti-cardiolipin antibodies), anti-β2GPI (anti-beta2-glycoprotein I), and LAC (lupus anticoagulant). It has been reported that the incidence of APS is approximately 5 new cases per 100,000 individuals per year, while the prevalence is estimated to be between 40 and 50 cases (1). APL, particularly anti-β2GPI, have been shown to promote the activation of monocytes, neutrophils, ECs, platelets, and trophoblasts, leading to the excessive expression and release of cytokines and chemokines, which can trigger thrombosis and contribute to other autoimmune and inflammatory complications (1).

The pathogenesis of APS follows the “second hit” model. APLs are necessary for the development of APS but are pathogenic only in the presence of a specific genetic background or secondary factors including infection, inflammation, surgery, prolonged bed rest, and oral estrogen-containing contraceptives (2). APLs interact with phospholipids or phospholipid-binding proteins leading to monocyte, neutrophil, endothelial cell, and platelet activation, which are involved in the activation of the NF-κB (nuclear factor kappa B) p38/MAPK (mitogen-activated protein kinases) signaling pathway and inhibition of Krüppel-like factors, leading to increased release of TF (tissue factor), IL(interleukin)-1, IL-8, TNF-α. This process involves binding to cell surface receptors such as TLR(toll-like receptor)2/4, apoER2 (apolipoprotein E receptor 2), and annexin A2. NETs induced by β2GPI/anti-β2GPI antibody complexes promote platelet aggregation and EC activation *in vitro*, further amplifying the procoagulant and proinflammatory phenotype. Pregnancy complications in OAPS can be attributed to the inhibitory effects of aPLs on EVT (extravillous trophoblast cell) invasion and proliferation as well as the immune dysregulation of the microenvironment at the maternal-fetal interface (3). In conclusion, aPLs are involved in the progression of APS by triggering a variety of mechanisms that lead to inflammation, thrombosis, and pathological pregnancy.

Given the susceptibility to concomitant cardiovascular events in patients with chronically positive antiphospholipid antibodies, identifying and controlling risk factors associated with thrombosis and/or adverse pregnancy outcomes is critical in clinical management. The European League Against Rheumatism (EULAR) in 2019 recommends the use of medications such as aspirin, vitamin K antagonists, heparin, and hydroxychloroquine, either alone or in combination, for adult patients with APS. In patients with catastrophic APS, intravenous immunoglobulin and plasma exchange are often considered (4). Despite aggressive treatment, some patients still experience multiple complications, which can be physically and emotionally taxing. The search for more effective treatments is therefore urgent.

**Figure 1:**
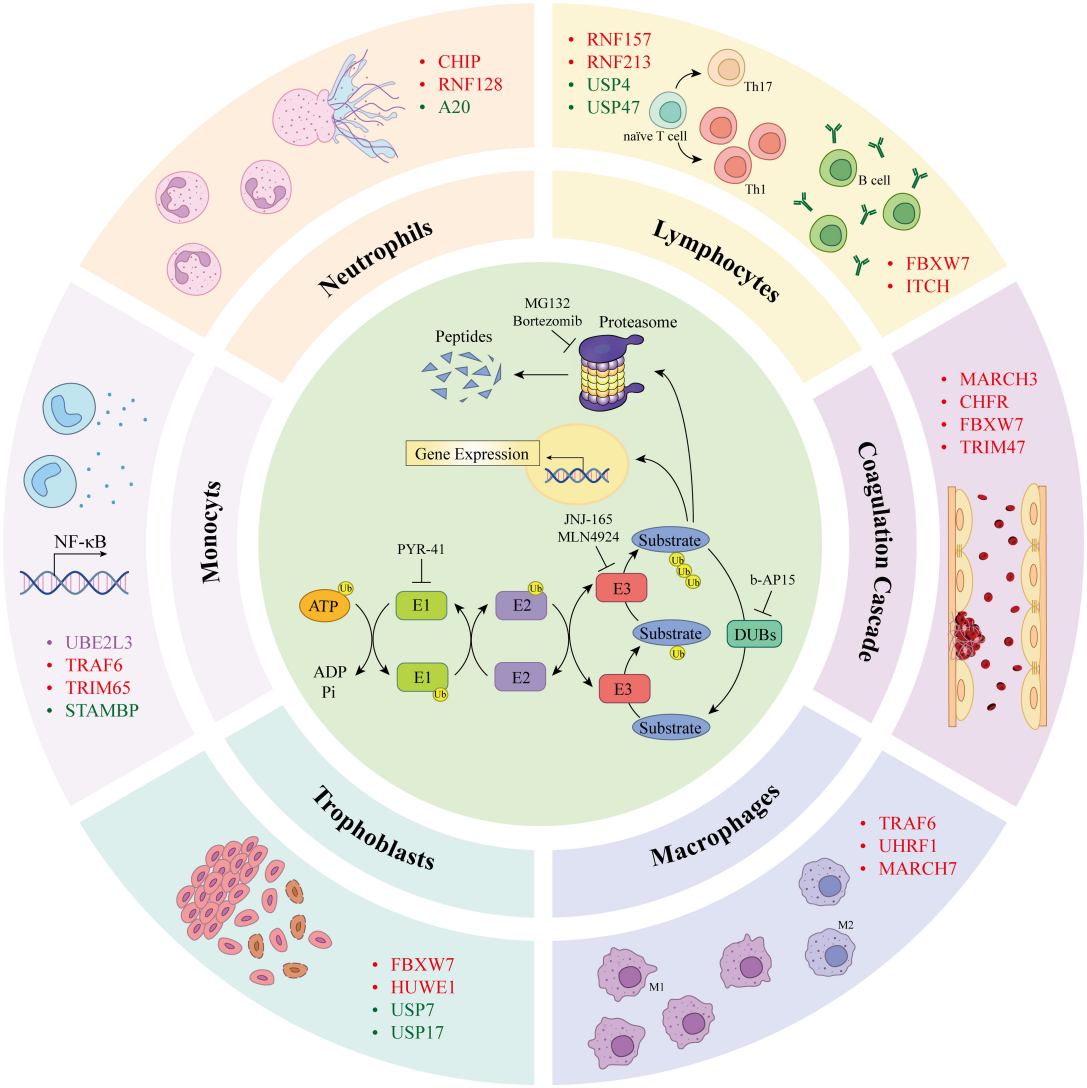
Components of the ubiquitin proteasome system (inner loop) are involved in the progression of the antiphospholipid syndrome (outer loop). Ubiquitin molecules are covalently bound to lysine residues of substrate proteins via a cascade reaction of E1 ubiquitin-activating enzymes, E2 ubiquitin-conjugating enzymes, and E3 ubiquitin ligases, leading to ubiquitination of the protein. Ubiquitinated proteins are degraded by the proteasome into peptides or participate in cellular signal transduction. Deubiquitinating enzymes reverse this process. PYR-41 and NSC697923 are inhibitors of E1 and E2 enzymes, respectively. JNJ-165 and MLN4924 are E3 ligases inhibitors frequently used to prevent protein ubiquitination; the inhibitor of DUBs, b-AP15, inhibits protein deubiquitination. MG132 and bortezomib are widely used proteasome inhibitors that suppress protein degradation via the proteasome pathway. Different UPS components modulate cellular functions associated with APS pathogenesis, including monocytes, neutrophils, lymphocytes, ECs, macrophages, and trophoblasts, potentially contributing to the promotion of APS pathogenesis. ATP, adenosine 5’-triphosphate; Ub, ubiquitin; AMP, adenosine monophosphate; Pi, phosphatidylinositol; UBE2L3, ubiquitin-conjugating enzyme E2 L3; TRAF4/6, TNF receptor associated factor 4/6; STAMBP, STAM-binding protein; CHIP, carboxyl terminus of Hsc70-interacting protein; RNF128, RING finger protein 128; A20, zinc finger protein A20; FBXW7, F-box/WD40 repeat-containing protein 7; ITCH, itchy E3 ubiquitin protein ligase; MARCH3, membrane associated RING-CH 3; CHFR, checkpoint with forkhead-associated and RING finger domains; YAP, yes-associated protein; LUBAC, linear ubiquitin chain assembly complex; UCHL1, ubiquitin carboxyl terminal hydrolase L1; OTULIN, OTU deubiquitinase with linear linkage specificity; UHRF1, ubiquitin like with PHD and RING finger domains 1; HUWE1, HECT, UBA And WWE domain containing E3 ubiquitin protein ligase 1; MARCH5, membrane associated RING-CH 5.

PTMs (protein translational modifications) increase the functional diversity of the proteome by covalently adding functional groups or proteins, hydrolyzing, cleaving regulatory subunits, or degrading proteins. Understanding PTMs is critical for advancing cell biology research and developing strategies for disease treatment and prevention. The UPS (ubiquitin-proteasome system) is a key post-translational modification mechanism that modulates a wide range of physiological processes, including cell survival, differentiation, inflammatory signaling, and autophagy (5). Ubiquitination refers to the covalent attachment of the small molecule protein ubiquitin to the lysine residue of the target protein, which is regulated by the coordinated action of three enzymes: E1 ubiquitin-activating enzymes, E2 ubiquitin-conjugating enzymes, and E3 ubiquitin ligases. Like other PTMs, ubiquitination is reversible and can be regulated by DUBs (deubiquitinases), which help maintain normal biological functions. Ubiquitin molecules have eight potential ubiquitination sites, including Met1, Lys6, Lys11, Lys27, Lys29, Lys33, K48 (Lys48), and K63 (Lys63), with K48 and K63 being the most extensively studied. It is widely recognized that K48-linked ubiquitin moieties signal proteasomal degradation, while K63-linked ubiquitination is crucial for DNA damage repair, immune response, and inflammation regulation (6). Emerging evidence suggests that UPS is instrumental in enhancing the secretion of inflammatory cytokines and autoimmune responses that are central to the development of autoimmune diseases (**Table 1**). The UPS is broadly involved in the regulation cellular functions and could serve as a valuable marker or therapeutic target for APS (**Figure 1**).

**Table 1:** The mechanisms of UPS components participating in the development of autoimmune diseases.

Disease	Altered Component	Type	Mechanism	Refs
SLE	UBE2L3	E2	Activating of NF-κB pathway via interaction with LUBAC	(92)
	TRIM21	RING E3	Suppressing the canonical NF‐κB pathway via monoubiquitination of phosphorylated IKKβ and subsequent autophagy	(93)
	FBXW7	SCF E3	Promoting cell apoptosis by catalyzing degradation of MCL1 through K48-linked ubiquitination	(94)
	SMURF1	HECT E3	Inhibiting the differentiation of naïve CD4 T cells towards Th17 and Th17.1 phenotype through the ubiquitination of RORγt	(95)
	USP8	USPs	Enhancing the cGAS-STING signaling pathway	(96)
	MYSM1	JAMMs	Suppressing the cGAS-STING pathway	(97)
	A20	OTU	Restricting B cell survival and averting the onset of autoimmune disorders via suppressing the NF-κB activation	(98)
RA	ZNRF3	RING E3	Influencing the level of TNF-α, IL-1β, and IL-6 in the collagen-induced arthritis mouse model	(99)
	TRIM18	RING E3	Encouraging synoviocyte proliferation and migration by inducing ubiquitin-mediated proteasomal degradation of DPP4, and augmenting the secretion of inflammatory cytokines	(100)
	USP5	USPs	Aggravating proinflammatory cytokines production and NF-κB signaling activation	(101)
APS	TRAF6	RING E3	Participating in TLR4/TF signaling pathway	(19)
	A20	OTU	Determining the activation threshold in dendritic cells, via modulation of canonical NF-κB activation	(102)
IBD	RNF180	RING E3	Reducing expression of ALKBH5 through ubiquitin-proteasomal pathway and disrupting Th17/Treg cell balance	(103)
	OTUD6A	OTU	Bounding to NACHT domain of NLRP3 inflammasome to enhance the stability of NLRP3, leading to increased IL-1β level	(104)
Psoriasis	UBE2L3	E2	Ameliorating psoriasis-like lesions and reducing pro-IL-1β and mature IL-1β levels in the epidermis	(105)
	RNF114	RING E3	A novel psoriasis susceptibility gene	(106)
	TRIM33	RING E3	Enhancing the inflammation by Anxa2/NF-κB pathway	(107)
	USP2	USPs	Promoting proliferation and inhibiting differentiation of keratinocytes	(108)
MS	RNF213	RING E3	Elevating nuclear translocation of FOXO1 mediated by K63-linked ubiquitination to orchestrate the differentiation of Tregs into CD4 T cells	(27)
	USP18	USPs	Lower USP18 gene expression levels associated with higher clinical disease activity	(109)
	UCHL1	UCHs	A potential candidate biomarker for MS diagnosis	(110)

SLE, systemic lupus erythematosus; UBE2L3, ubiquitin-conjugating enzyme E2 L3; LUBAC, linear ubiquitin chain assembly complex; TRIM21, tripartite motif containing 21;IKKβ, inhibitor of kappa B kinase β; FBXW7, F-box and WD repeat domain-containing 7; SCF, SKP1-CUL1-F-box; MCL1, myeloid cell leukemia-1; SMURF1, Smad ubiquitination regulatory factor 1; RORγt, retinoic acid-related orphan receptor gamma t; cGAS-STING, cyclic GMP-AMP synthase-stimulator of interferon genes; MYSM1, Myb-like, SWIRM, and MPN domains 1; A20, zinc finger protein A20; OUT, ovarian tumor-related protease; RA, rheumatoid arthritis; ZNRF3, zinc and RING finger 3; TNF-α, tumor necrosis factor-α; IL-1β, interleukin-1β; TRAF6, TNF receptor associated factor 6; TLR4/TF, toll-like receptor 4/tissue factor; IBD, inflammatory bowel disease; RNF180, RING finger protein 180; ALKBH5, Alk B homologue 5; IκBα, recombinant inhibitory subunit of NF-κB alpha; OTUD6A, ovarian tumor deubiquitinase 6A; NACHT, nucleotide-binding and oligomerization domain; NLRP3, NOD-like receptor thermal protein domain associated protein 3; Anxa2, annexin A2; MS, multiple sclerosis; FOXO1, forkhead box protein O1; UCHL1, ubiquitin carboxyl terminal hydrolase L1.

Notably, the activation of the NF-κB pathway, which is highly associated with inflammatory responses and coagulation disorders in APS, is regulated by the UPS (7). The NEMO (NF-κB essential modulator), IKK1 (inhibitor of NF-κB kinase 1), and IKK2 constitute the IKK complex. Linear polyubiquitination of LUBAC (linear ubiquitin chain assembly complex)-regulated NEMO is essential for NF-κB activation (8). Linear diubiquitin-activated IKK2 phosphorylate IκBα (NF-κB inhibitor-α) and NF-κB p105 subunits, thereby activating the NF-κB and ERK pathways and triggering the release of TNF-α, IL-1β, IL-8, and TF. Conversely, the deubiquitinating enzymes OTULIN (OTU deubiquitinase with linear linkage specificity), A20 (zinc finger protein A20), and CYLD (cylindromatosis) can reverse this signaling pathway, thereby attenuating the expression of downstream molecules (9). The induction of NF-κB translocation to the nucleus by anti-β2GPI is analogous to that induced by TNF-α. TNF-α induces a prothrombotic phenotype in ECs and activates the complement system, thereby accelerating thrombosis in synergy with factor Xa. In the aPLs-treated mice model demonstrates that levels of TNF-α in placenta correlate with recurrent miscarriage (10). TNF-α binds to its cell-surface receptor, TNFR1, to trigger the assembly of protein complexes, which primarily signal to activate MAPK and NF-κB-dependent genes. This process is also regulated by linear ubiquitination (11). Furthermore, dysregulation of ubiquitination associated with the NF-κB pathway results in cell death. This further indicates that proteins such as LUBAC and A20 are involved in the regulation of rheumatic diseases (12). In brief, targeting the UPS may modulate the NF-κB signaling pathway, thereby slowing the progression of APS.

To deepen our understanding of UPS-mediated pathology of APS, this review aims to provide a comprehensive overview of UPS involvement in APS progression and UPS-mediated dysfunction of monocytes, neutrophils, ECs, platelets and trophoblasts, while also discussing the potential of targeting the UPS for diagnostic and therapeutic purposes in APS.

## 2 UPS in inflammatory cells

### 2.1 Monocytes

APL are known to activate monocytes, leading to the expression of TF, IL-1β and TNF-α, thus triggering a cascade of thrombosis, inflammation and autoimmune responses, primarily through the NF-κB signaling pathway. Studies have demonstrated that the UPS in monocytes have an obligatory role in regulating pathogenesis of APS (13) (**Table 2**). Mononuclear cells isolated from patients with the APS exhibit increased expression of caspase-1 and NLRP3 indicating the chronic inflammation activation in APS patients.

**Table 2:** The mechanisms by which the UPS contributes to APS progression in vital cells.

Cells	Altered Component	Type	Mechanisms	Refs
Monocytes	UBE2L3	E2	UBE2L3 is the preferred E2 for LUBAC *in vivo*, and inhibition of UBE2L3 attenuates IκBα phosphorylation	(14)
	TRAF6	RING E3	Activating NLRP3 and promoting IL-1β and IL-18 secretion	(15)
	TRIM65	RING E3	Degrading NLRP3 inflammasome and inhibiting their activation through ubiquitination	(17)
	STAMBP	JAMM	Knockdown of STAMBP enhances NLRP3 activity by increasing K63-linked polyubiquitination of NLRP3	(18)
Neutrophils	RING128	RING E3	Promoting ubiquitination degradation of TLR4	(22)
	A20	OTU	Repressing NF-κB signaling by deubiquitinating enzyme activity	(23)
T cells	RNF157	RING E3	Regulating Th differentiation by ubiquitinating HDAC	(26)
	RNF213	RING E3	Improving K63-linked ubiquitination of FOXO1 thereby specifically regulating Treg cell differentiation and increasing anti-inflammatory factor IL-10 production	(27)
	USP4	USPs	Stabilizing the transcription factor RORγt and stimulating Th17 cell differentiation	(28)
	USP47	USPs	Suppressing YTHDF1-mediated c-Myc translation	(29)
B cells	FBXW7	SCF E3	Maintaining B-cell maturation and homeostasis, and FBXW7 deficiency may lead to the accumulation of substrate proteins and thus affect the function of germinal center	(32)
	ITCH	HECT E3	Ubiquitinating eIF3a, eIF3c, and eIF3h and limiting production of antibodies	(33)
	Peli1	RING E3	Mediating NF-κB-inducing kinase K48 ubiquitination and degradation	(34)
	A20	OTU	A20 deficiency in B cells stimulates cellular autoantibody production	(35)
ECs	MARCH3	RING E3	Ubiquitinating occludin and disrupting the endothelial barrier	(38)
	CHFR	RING E3	Involving in ubiquitination and degradation of VE-cadherin	(39)
	FBXW7	SCF E3	Enhancing KLF2 ubiquitination and degradation via the proteasome	(42)
			Degrading KPNA2 to block its interaction with transcription factor p65 and interferon regulatory factor 3	(43)
	TRIM47	RING E3	Elevating K63-Linked ubiquitination of TRAF2, therefore suppressing NF-κB and MAPK signaling pathways and up-regulates endothelial cell inflammatory factor expression	(44)
Platelets	SHARPIN	Component of LUBAC	Interacting with the cytoplasmic tail of platelet-specific αIIbβ3 to activate NF-κB	(49)
	RNF181	RING E3	Binding to the cytoplasmic tail of platelet-specific αIIbβ3 to stimulate platelet aggregation and thrombosis	(50)
Trophoblasts	FBXW7	SCF E3	Reducing cyptochrome circadian regulator 2 protein via UPS, which in turn regulating the proliferation and migration of trophoblast cells	(60)
	HUWE1	HECT E3	Associating with DOCK1 to maintain DUSP4 protein stability, and increasing the ability of migration through activation of the ERK pathway	(61)
	USP7	USPs	Supporting trophoblast invasive capacity through the EZH2/Wnt/β-catenin signaling pathway	(62)
	USP17	USPs	Overexpression of USP17 enhances HDAC2 stability and expression levels, thereby augmenting STAT1 activity and inhibiting NF-κB signaling	(63)
	USP14	USPs	Inducing NF-κB signaling and boosting IL-1β expression	(64)
	USP22	USPs	USP22 mediates ADAM9 deubiquitination, which interacts with ADAM9 to regulate the Wnt/β-catenin pathway	(65)
	LMP2	Proteasome subunit	Triggering p50 production and nuclear translocation of NF-κB to increase MMP-2 and MMP-9 expression	(66)
Macrophages	TRAF6	RING E3	Interconnecting with AOC4P and impeding M2 macrophage polarization	(67)
	UHRF1	RING E3	Reducing UHRF1 promotes macrophage polarization to M1-type and secretion of CXCL2 and IL-1β via activation of the MyD88/NF-κB pathway	(55)
	MARCH7	RING E3	Mediating NLRP3 ubiquitination and thus attenuating macrophages polarization toward the M1-type	(68)

UBE2L3, ubiquitin-conjugating enzyme E2 L3; TRAF6, TNF receptor associated factor 6; TRIM, tripartite motif; STAMBP, STAM-binding protein; JAMM, Jab1/MPN domain associated metalloisopeptidase; RING, really interesting new gene; A20, zinc finger protein A20; RNF, RING finger protein; USP, ubiquitin-specific protease; FBXW7, F-box/WD40 repeat-containing protein 7; ITCH, itchy E3 ubiquitin protein ligase; MARCH, membrane associated RING-CH; CHFR, checkpoint with forkhead-associated and RING finger domains; SHARPIN, shank associated RH domain interactor; HUWE1, HECT, UBA And WWE domain containing E3 ubiquitin protein ligase 1.

The UPS plays a pivotal role in controlling inflammatory signals by modulating the activity of NLRP3 and NF-κB pathway. UBE2L3 (ubiquitin-conjugating enzyme E2 L3), an E2 ubiquitin-conjugating enzyme, is essential for the LUBAC-mediated activation of NF-κB in monocytes (14). The anti-β2GPI/β2GPI complex has been shown to simultaneously and acutely enhance mRNA levels of TRAF6 (TNF receptor associated factor 6) and TRAF4. It has been demonstrated that TRAFs, which possess E3 ligase activity, play a role in the activation of the NLRP3 inflammasome, which is crucial for regulating the TLR4/TF signaling pathway in activated THP-1 cells (15). The TRIM (tripartite motif) proteins are a versatile family of E3 ligases, are composed of over 80 distinct members in human and are recognized for their roles in antiviral responses. Numerous studies have demonstrated the regulatory roles of TRIM proteins in mediating inflammation (16). TRIM65 has the ability to mediate NLRP3 ubiquitination, thereby inhibiting caspase-1 activation and IL-1β secretion, which negatively regulates inflammatory responses in monocytes (17). J. S. Bednash et al. (18) identified STAMBP (STAM-binding protein), a JAMM (Jab1/MPN domain associated metalloisopeptidase) metalloprotease in the DUBs family, as a negative regulatory factor that helps maintain the inflammatory balance. Cellular depletion of STAMBP increases NLRP3 K63 chain polyubiquitination, resulting in enhanced NLRP3 inflammasome activation. The specific proteasome inhibitor MG132 can attenuate the expression of TRAFs and TF that are induced by anti-β2GPI/β2GPI complex (19). In brief, the UPS plays a significant role in the anti-β2GPI/β2GPI-stimulated TLRs signaling pathway in THP-1 cells and contributes to the pathological processes of APS. These findings provide a robust foundation for the development of targeted therapies for APS.

### 2.2 Neutrophils

Neutrophils constitute the majority of white blood cells in peripheral blood, accounting for approximately 50% to 70% of the total, and they play a critical role in immune responses, particularly in the context of APS. Anti-β2GPI can stimulate neutrophils to release NETs (neutrophil extracellular traps), which are extracellular web-like scaffolds of decondensed chromatin adorned with microbicidal proteins such as PAD4 (peptidylarginine deiminase 4) and MPO (myeloperoxidase). NETs have capacity to initiate thrombosis *in situ* through various mechanisms, including activation of coagulation factors, the endothelium and platelets, thereby exacerbating autoimmune diseases (1). Meanwhile, APS patients experience increased NETs generation accompanied by decreased clearance, leading to acute thrombosis, occlusive vascular disease, and adverse pregnancy outcomes (20).

Research on NETs has primarily focused on their main components and regulatory mechanisms. For instance, ALDH2 (aldehyde dehydrogenase 2) has been found to inhibit NETosis by promoting K48-linked polyubiquitination and degradation of PAD4, thus maintaining innate immune system homeostasis. Although ALDH2 is not a component of the UPS, it can facilitate the binding of PAD4 to the E3 ubiquitin ligase CHIP (carboxyl terminus of Hsc70-interacting protein). Pharmacological activation of ALDH2 using Alda-1 has been shown to significantly alleviate septic acute respiratory distress syndrome by inhibiting NETs (21). *In vitro* studies have demonstrated that the RING (really interesting new gene) finger protein RNF128 (RING finger protein 128) decreases the level of MPO, restrains NF-κB signaling, and reduces the expression of proinflammatory cytokines such as TNF-α, IL-1β, and IL-6, thereby monitoring neutrophil activation (22). A20 is a cytoplasmic zinc finger protein that inhibits NF-κB activity through its deubiquitinating activity, which is vital for limiting inflammation in neutrophils. Research has revealed that mutant A20 cells exhibit heightened inflammatory characteristics, as evidenced by increased levels of IL-1β, IL-9, and IL-17A (23). *A20 C103A* mutation or *A20 rs2230926* polymorphism has been associated with upregulation of PADI4 expression. Consequently, *A20 C103A* cells display enhanced protein citrullination and NETs formation, thereby exacerbating susceptibility to SLE. Mutations in the A20 locus have been linked to a variety of autoimmune diseases, including RA, psoriasis, SLE, celiac disease, Crohn’s disease and diabetes, likely through the deregulation of the NF-κB pathway (5).

In brief, the role of ubiquitination and neutrophils in the pathogenesis of autoimmune diseases has attracted considerable scholarly attention (**Table 2**). Although advancements have been achieved, there is an imperative need for further in-depth research to elucidate the intricate interactions between neutrophils and the UPS in APS, which is fundamental to precisely designing targeted treatments and improving the overall prognosis for patients.

### 2.3 Lymphocytes

#### 2.3.1 T cells

Autoreactive CD4 T cells against β2GPI are crucial in the production of aPL and the progression of clinical manifestations in APS. A recent retrospective study involving 67 patients with APS and 40 healthy controls revealed patients with APS had an immune disorder, characterized by elevated Th1, reduced anti-inflammatory Tregs levels and an increased Th17/Treg ratio (24). Over the past decades, research has firmly established the significance of protein ubiquitination in regulating T cell-mediated immunity (**Table 2**). Various E3 ligases and DUBs have been identified as pivotal regulators of T cell function, influencing both positive and negative aspects of immune responses (25). RNF157 is an important regulator of CD4 T cell differentiation. It promotes the differentiation of Th1 cells, which are associated with cellular immunity and inflammation, while also reducing the differentiation of Th17 cells and the expression of chemokine receptors CCR4 (C-C chemokine receptor type 4) and CXCR3 (C-X-C motif chemokine receptor 3) in CD4 T cells (26). Besides, RNF213 interacts with FOXO1 (forkhead box protein O1) and facilitates the nuclear translocation of FOXO1 through K63-linked ubiquitination, thereby specifically promoting the differentiation of Treg cells and attenuating the development of autoimmune diseases. *In vivo*, RNF213-deficient mice (*Rnf213* ^-/-^) are observed to exhibit increased serum levels of the pro-inflammatory cytokines IFN-γ, IL-17A, and GM-CSF (granulocyte-macrophage colony-stimulating factor), alongside decreased secretion of the anti-inflammatory cytokine IL-10 (27). Targeting DUBs also presents a promising avenue for modulating T cell responses. USP4 (ubiquitin-specific protease 4) is responsible for Th17 cell differentiation by stabilizing the transcription factor RORγt (retinoic acid-related orphan receptor gamma t). Inhibition of USP4 may offer a valuable therapeutic target for treating Th17-dependent autoimmune diseases, such as multiple sclerosis or RA (28). Another member of the USP family, USP47, orchestrates Treg homeostasis in an m6A-dependent manner, suggesting novel approaches for immunomodulation of autoimmune diseases by targeting USP47 (29).

#### 2.3.2 B cells

B cells contribute to the pathogenesis of autoimmune disease primarily through the production of antibodies (30). APS patients with clinical manifestations of venous thromboembolism displayed B lymphocyte disturbances as evidenced by increased proportions of B1 cells and naïve B cells, while memory B cells were reduced (31). The UPS is known to regulate B cell differentiation and antibody production (**Table 2**). FBXW7 (F-box/WD40 repeat-containing protein 7) is highly expressed in B1 cells and involved in the regulation of antibody responses. The ablation of FBXW7 has been demonstrated to ameliorate pathogenesis in a model of autoimmune disease, specifically collagen-induced arthritis, by reducing the production of autoantibodies (32). The E3 ligase ITCH (itchy E3 ubiquitin protein ligase) is another critical regulator that maintains antibody levels and prevents autoimmune disease in both humans and mice by limiting the metabolism and proliferation of naïve B cells (33). Peli1 has a B-cell-intrinsic function that protects mice against lupus-like autoimmunity. Within B cells, Peli1 negatively regulates NF-κB inducing kinase, by mediating its ubiquitination via K48. A deficiency of Peli1 in B cells leads to autoantibody production through non-canonical NF-κB signaling (34). Additionally, the inactivation of A20 results in constitutive NF-κB activation in human B-cells. A20^B-KO^ (B cell-specific ablation) mice exhibit significantly elevated serum IgM, IgG2a, and IgA levels compared to wild-type mice and demonstrated an increased number of effector-type T cells, possibly as a result of impaired crosstalk between T cells and A20-deficient B cells (35). Taken together, these data suggest that E3 ligases in B cells may be attractive targets for the development of new therapies for autoimmune diseases.

## 3 UPS in coagulation cascade

Dysfunction of natural anticoagulant systems, mediated by antiphospholipid antibodies, is central to the pathogenesis of APS. ECs dysfunction, coagulation activation and depressed fibrinolysis are recognized thrombogenic pathways of primary APS. Endothelium-derived nitric oxide, produced by endothelial nitric oxide synthase, is important for maintaining normal endothelial function and vascular health (36).

### 3.1 Endothelial barrier

Emerging research suggests that the UPS is intricately involved in endothelial barrier function, endothelial activation, cell apoptosis, and autophagy (37). Disruptions in endothelial barrier function not only boost platelet adhesion and aggregation but are also correlated with inflammation in APS. Here we focus on the role of ubiquitin and deubiquitination events in ECs implicated in APS pathophysiology (**Table 2**).

MARCH3 (membrane associated RING-CH 3), a key enzyme identified through siRNA library screening, is essential for preserving endothelial permeability. MARCH3 silencing in ECs exacerbates cell-cell contacts and disrupts the endothelial barrier, as evidenced by the upregulation of the tight junction-encoding gene OCLN (occludin) (38). Another E3 ligase CHFR (checkpoint with forkhead-associated and RING finger domains), mediates ubiquitylation-dependent degradation of VE-cadherin, which is vital for maintaining vascular integrity and endothelial homeostasis. Both processes involve the activation of the FoxO signaling pathway (38, 39). Additionally, the deubiquitinating enzyme UCHL1 (ubiquitin carboxyl terminal hydrolase L1) has been shown to have a protective effect in models of increased pulmonary vascular permeability. Knockdown of UCHL1 or its pharmacological inhibition LDN results in an increase in vascular leakage both *in vitro* and *in vivo* (40). Endothelial barrier compromise results in augmented expression of adhesive molecules and intensified leukocyte adhesion, consequently precipitating an exacerbated inflammatory cascade and the pathogenesis of thrombosis.

### 3.2 Endothelial function

Tight regulation of cell death and NF-κB responses in the ECs is important for homeostasis and pathology of the immune system, as demonstrated in genetic mouse models and in patients with APS (20). YAP (yes-associated protein) promotes the ubiquitination and proteasomal clearance of TRAF6 to inhibit the activation of NF-κB signaling. Consuming TRAF6 in ECs can rescue the inflammatory phenotype observed in endothelial-specific YAP knockout mice (41). Another RING-type E3 ligase, FBXW7, is involved in regulating ECs function as well. It recognizes phosphorylation degradation domains on KLF2 (krüppel-like factor 2), leading to KLF2 ubiquitination and subsequent degradation via the 26S proteasome pathway (42). FBXW7 can also degrade KPNA2 (karyopherin subunit alpha 2) through ubiquitination, thereby alleviating endothelial dysfunction and inflammatory responses by inhibiting p65 and interferon regulatory factor 3 (43). TRIM47, a novel ECs modulatory factor, has been shown to augment inflammation and monocyte adhesion when overexpressed, manifested as a significant upregulation in the levels of IL-1β, IL-6, and IL-8 mRNA and a potentiation of ICAM-1 (intercellular cell adhesion molecule-1), VCAM-1 (vascular cell adhesion molecule-1), E-selectin, and MCP-1 (monocyte chemotactic protein-1). Mechanistically, TRIM47 facilitates K63-linked ubiquitination of TRAF2 thus activating NF-κB and MAPK signaling pathways (44).

MDM2 (murine double minute 2) is an E3 ligase containing a RING domain, which acts as a negative regulatory factor of p53 protein. MDM2 binds to p53, inhibiting its interaction with DNA and promoting its monoubiquitination and nuclear export, or facilitating its polyubiquitination and degradation via the proteasome (45). Dysregulated MDM2 is implicated in cardiovascular impairments as well such as atherosclerosis. In endothelial dysfunction and atherosclerosis associated with APS-related features, overexpression of MDM2 exacerbates mitochondrial damage and activates TLR9/NF-κB and NLRP3/caspase-1 pathways (46). MDM2 is a recently identified E3 ubiquitin ligase, regulates endothelial function by modulating mitochondrial energy metabolism. APLs purified from the serum of APS patients with thrombosis and pregnancy complications increase mitochondrial hyperpolarization in ECs, thereby supporting a mechanism by which aPLs induce oxidative stress (47). When mitochondria are damaged, elevated mitochondrial reactive oxygen species contributes to the release of mitochondrial DNA, which in turn activates the TLR9-MyD88-NF-κB pathway, induces inflammasome activation, and exacerbates the inflammatory response (47). To summarize, these findings support the link between the UPS and mitochondrial dysfunction in APS, offering a new perspective on the mechanisms underlying rheumatic diseases.

### 3.3 Platelets

Anti-β2GPI bind to platelets to promote the release of procoagulant factors, including thromboxane B2, TF, and platelet factor 4, which in turn promote thrombotic manifestations and pregnancy loss. Most importantly, the UPS is a key participant in maintaining platelet homeostasis (**Table 2**). Resting platelets exhibit extensive ubiquitination, with 1,634 ubiquitylated peptides derived from 691 proteins and over 900 of these peptides showing an increase of more than twofold following stimulation with collagen-related peptide. Multiple sites of ubiquitylation were identified on spleen tyrosine kinase and FcRγ (Fc receptor γ) chain, which are involved in the glycoprotein VI signaling pathway. Collagen-related peptide triggers platelet adhesion and dense granule secretion indicating that ubiquitination plays a role downstream of platelet collagen receptor glycoprotein VI (48). The adapter molecule SHARPIN which is also expressed by human platelets can directly bind to the cytoplasmic tail of αIIbβ3 and promote the activation of the NF-κB pathway as part of the Met-1 LUBAC. Mice with platelet-specific deficiency of SHARPIN exhibit prolonged bleeding time (49). α-subunit KVGFFKR motif exerts a vital influence on the regulation of integrin activation. RNF181, an emerging platelet protein with E3 ligase capabilities, interacts directly with the platelet-specific αIIbβ3 cytoplasmic tail via its highly conserved KVGFFKR regulatory motif, which is implicated in platelet aggregation and thrombotic disorders (50). Deubiquitinating enzymes USP14 and UCHL5, expressed by platelets, play a role in platelet function. Inhibition of deubiquitination reduces platelet adhesion and impairs occlusive thrombosis. More recent next-generation RNA-sequencing and proteomic profiling studies confirmed that platelets express key 20S and 26S proteasomal components, perform chymotrypsin-, trypsin-, and caspase-like proteolytic activities which largely ascribe to the proteasome a regulatory role in platelet production, viability, and function (51).

### 3.4 Antithrombin

Individuals with APS frequently present with diminished levels or a dysfunction of antithrombin, a circumstance that can precipitate clot formation (52). Capitalizing on its capacity to bind with AT and enhance anticoagulant efficacy exponentially, heparin is routinely utilized to manage and preempt thrombotic episodes in APS and analogous thrombotic disorders (53). Cullin2 and USP2 have been identified as novel regulators of antithrombin expression. Cullin2 and its interacting partner RBX1 are associated with antithrombin, leading to its ubiquitination and protease-dependent clearance, while USP2 overexpression inhibits this ubiquitination. USP2 inhibitor ML364 is capable of promoting antithrombin degradation (54). The role of the ubiquitin proteasome system in the coagulation cascade still needs further research to elucidate its detailed mechanism.

## 4 UPS in pregnant morbidity

The differentiation and migration of trophoblast cells, as well as the remodeling of maternal blood vessels, are crucial for the establishment and maintenance of pregnancy (55). Human decidual macrophages present in the interstitium of placental villi participate in shaping the immune microenvironment, contributing to immune tolerance. Disruption of trophoblast function or the immune microenvironment can lead to placental developmental defects and pregnancy complications such as recurrent miscarriage, preeclampsia, and intrauterine growth restriction.

Specifically, trophoblast cells express β2GPI, and anti-chorionic phospholipid antibodies work by reducing the proliferation and invasion of extra-embryonic trophoblasts and triggering inflammation at the maternal-fetal placental interface, collectively impairing placental formation. The Wnt/β-catenin signaling pathway has been demonstrated to promote trophoblast cell proliferation, invasion, and migration. β-catenin translocates into the nucleus, thereby inducing the expression of downstream genes such as MMPs (matrix metalloproteinases), and alleviating pathological pregnancies (56). The interaction of ApoER2 with β2GPI is required for aPLs-induced trophoblast dysfunction, which involves the inhibition of EGR-induced Akt phosphorylation (57, 58).

Imbalances in the polarization of decidual macrophages have been identified as a key factor contributing to adverse pregnancy outcomes. Trophoblast-derived lactate has been shown to regulate M1 and M2 macrophage polarization via glycolysis and oxidative phosphorylation during early pregnancy. Under conditions of extreme hypoxia (1-3%), elevated lactate levels promote M1 polarization through activation of the HIF-1α/SRC/LDHA pathway, thereby mediating the development of pregnancy complications. Using single-cell sequencing of early gestational decidual tissue, C. Lu et al. (59) identified a significant increase in macrophages characterized by glycolysis as the main metabolic feature, thereby confirming the increased polarization of M1-type macrophages in OAPS.

### 4.1 Trophoblasts

The downregulation of long non-coding RNA MALAT1 is associated with miscarriage, as well as a reduction in trophoblast cell proliferation and invasiveness. MALAT1 recruits FBXW7 to trigger the ubiquitin-mediated degradation of cyptochrome circadian regulator 2 protein, while overexpression of cyptochrome circadian regulator 2 significantly inhibits trophoblast cell migration and invasion by repressing the MMP2/9 (matrix metalloproteinase 2/9). Scaffold protein CUL1 of FBXW7 ubiquitin ligase complex has the ability to promote the proliferation and migration of trophoblast cells, whose downregulation has been observed in PE (preeclampsia) placental tissue (60) (**Table 2**). Similarly, Dedicator of cytokinesis 1 is downregulated in placental villi of patients with recurrent spontaneous abortion. DOCK1 participates in regulating DUSP4 (dual specificity phosphatase 4) ubiquitin levels through the E3 ligase HUWE1 (HECT, UBA And WWE domain containing E3 ubiquitin protein ligase 1), and its expression determines the invasive characteristics of EVT (61).

The deubiquitinating enzyme USP7 regulates trophoblast cell functions by interacting with EZH2 (enhancer of zeste homolog 2) and modulating the Wnt/β-catenin signaling pathway. In the villous tissue of women with recurrent miscarriage, decreased USP7 expression is associated with reduced trophoblast cell invasion and migration (62). USP17 is significantly downregulated in PE patients. Overexpression of USP17 promotes proliferation, migration, and invasion of the trophoblast layer. Upregulation of USP17 enhances the level of HDAC2 protein, strengthens the interaction between HDAC2 and signal transducer and activator of transcription 1, resulting in increased STAT1 activity and inhibition of NF-κB signaling (63). Conversely, the expression of USP14 in placental tissue of patients with preeclampsia was significantly upregulated, knocking down or inhibiting USP14 significantly eliminated upregulation of NF-κB activation and production of pro-inflammatory cytokines (TNF-α and IL-1β) (64). In PE, USP22 mRNA was highly elevated and removes ubiquitination on ADAM9 (a disintegrin and metalloprotease 9) and increasing its stability, therefore weakening trophoblast cell proliferation and invasion (65).

Anti-β2GPI antibodies can inhibit the secretion of MMP2 and MMP9 by human trophoblast cells, suggesting essential roles in obstetric APS. Large multifunctional peptidase 2 (LMP2), a proteasome subunit critical for proteasome activity, influences the activity of MMP2 and MMP9, which are zinc-dependent proteases that degrade the extracellular matrix and are implicated in inflammation, trophoblast invasion, and vascular remodeling during pregnancy. Mechanistically, LMP2 contributes to the degradation of NF-κB inhibitor α and the generation of p50. Inhibiting LMP2 can block the transfer of active NF-κB heterodimers into the nucleus, thereby inhibiting the expression and activity of MMP (66).

### 4.2 Macrophages

Upon the invasion of EVT into the decidua, macrophages tend to adopt the M2 phenotype, which is essential for pregnancy maintenance. An imbalance between the M1 and M2 macrophage phenotypes can lead to adverse pregnancy outcomes in APS. Single-cell sequencing transcriptome analysis has revealed that in obstetrics APS patients, macrophages with heightened glycolytic activity are more prevalent within the decidual immune cell population (59). Macrophage polarization is regulated by UPS (**Table 2**). Notably, S. Wu et al. (67) discovered an abnormal increase in the AOC4P (amine oxidase copper containing 4 pseudogene) of amine oxidase in the villi of RSA (recurrent spontaneous abortion) patients. AOC4P interacts with TRAF6, leading to the upregulation of TRAF6 expression and promoting M1 macrophage polarization, which could be implicated in the pathogenesis of pregnancy morbidity (67). Knockdown of the E3 ubiquitin ligase MARCH7 reduces the ubiquitination levels of NLRP3, which contributes to the subsequent assembly of NLRP3 inflammasome and promotes macrophage polarization toward the M1-type (68). UHRF1 (ubiquitin like with PHD and RING finger domains 1) with E3 ligase function can promote M1 macrophage polarization by activating the MyD88/NF-κB signaling pathway and CXCR2/IL-1R1 receptor, further influencing pregnancy outcomes (55). Interestingly, TGF-β secreted by M2 macrophages, can induce trophoblast migration and invasion, while anti-TGF-β antibodies can mitigate this process (69). TGF-β also upregulates the expression of USP2a in trophoblast cells, interacts with TGFBR1, and promotes the transcription of epithelial-mesenchymal transition-related genes. USP2a activates the PI3K/Akt/GSK3β signaling pathway, facilitating the nuclear translocation of β-catenin and activating EMT in trophoblast cells (69). In conclusion, the UPS plays a significant role in the pathological mechanisms of obstetric APS, partly by influencing macrophage polarization. Targeted alteration of macrophage polarity may be a future therapeutic direction for OAPS.

## 5 Therapies targeting the UPS

The ubiquitin-dependent pathway is a pervasive cellular mechanism that influences myriad aspects of cell biology, prompting the development of molecules capable of inhibiting, activating and/or modulating the mechanisms of the ubiquitin pathway as research and therapeutic tools. A notable therapeutic success is the combination therapy employing the proteasome inhibitor bortezomib with the E3 ligase-targeting immunomodulatory drugs thalidomide and lenalidomide, which has revolutionized the treatment of multiple myeloma (70). The UPS has been implicated in the pathogenesis of autoimmune diseases. This association has shed light on the complex pathological mechanisms underlying APS and has unveiled new therapeutic targets (71).

### 5.1 Inhibitors targeting the UPS

Low-dose proteasome inhibitors are beneficial for the anti-inflammatory effects on vascular cells both *in vitro* and *in vivo*, and these effects are primarily attributed to the suppression of NF-κB activation. Researchers found that after incubating HUVECs with low concentrations of proteasome inhibitors MG132 (70nM) or MG262 (4nM) for 24 hours, this results in a reduction of reactive oxygen species and VCAM-1 production, consequently diminishing monocyte-HUVEC adhesion. Furthermore, low-dose proteasome inhibitors enhance the expression and activity of eNOS, thereby improving endothelial function. The use of proteasome inhibitors can significantly reduce IgG-APS-induced thrombus size in mice and inhibit TF upregulation in peritoneal macrophages and carotid artery homogenate (72). Bortezomib’s therapeutic potential extends beyond oncology, as evidenced by its capacity to exert beneficial anti-inflammatory and antithrombotic effects and mitigate the progression of atherosclerotic lesions in a murine model with low-density lipoprotein receptor deficiency when administered at low doses. The anti-inflammatory and antithrombotic properties of proteasome inhibitors could be harnessed for the treatment of APS (73).

Ubiquitin-activating enzyme E1 is located at the peak of the ubiquitin cascade reaction, and targeting E1 could influence subsequent enzymatic steps. PYR-41 is an irreversible, cell permeable inhibitor of ubiquitin-activating enzyme E1 UBA1 (ubiquitin-activating enzyme E1), which has been reported to inhibit the expression of IgE, IFN-γ and also reduced ear thickening and skin damage in mouse models of atopic dermatitis, associated with the inhibition of the NF-κB signaling pathway (74). The ubiquitin binding enzyme E2 determines the specific connection mode and length of the ubiquitin chain, and targeting E2s provides more selectivity compared to targeting E1 enzymes. Several E2 enzymes, including UBE2Q2, UBE2S, UBE2T2, UBEH9, UBEH10, and UBCH13, have been directly implicated in human disorders and cellular proliferation, further validating them as therapeutic targets. Notably, UBCH13 enhances NLRP3 inflammasome activation by promoting site-specific K63 ubiquitination of NLRP3. The small molecule inhibitor NSC697923 of UBCH13 inhibits NLRP3 inflammasome activation by suppressing NF-κB signaling, thereby alleviating the inflammatory response (75).

Treatment with MDM2 inhibitors JNJ-165 has been shown to alleviate oxidized low-density lipoprotein-induced mitochondrial damage and the production of pro-inflammatory cytokines in ECs (76). The NF-κB-antagonistic and p53-agonistic activities of MDM2 inhibitors elicit potent therapeutic effects in experimental lymphoproliferative autoimmune disorders such as SLE and APS (77). The novel role of MDM2 in vascular inflammation and mitochondrial bioenergetics presents a promising intervention target for the prevention and treatment of inflammation-related diseases. IL-17 is involved in chronic inflammation occurring during the pathogenesis of inflammation and autoimmune diseases. Significantly elevated IL-17 concentrations have been observed in primary APS patients who also exhibit thrombocytopenia, potentially contributing to the vascular manifestations of primary APS (78). Upon stimulation with IL-17A, the adaptor protein Act1 is recruited to IL-17R, subsequently recruiting the E3 ubiquitin ligase TRAF6 and TAK1, thereby initiating downstream signaling events, including the activation of MAPK and NF-κB pathways. MLN4924 has potential to attenuate IL-17A-induced autoimmune diseases by reducing the secretion of various inflammatory cytokines, including IL-1β, IL-6, TNF-α, and MCP-1. Mechanistically, MLN4924 promotes the ubiquitination and degradation of Act1, disrupting its interaction with IL-17R and impeding the formation of the IL-17R/Act1/TRAF6 complex, resulting in decreased expression of pro-inflammatory factors (79).

Several DUBs have emerged as potential targets for modulating inflammation. b-AP15, a DUB inhibitor targeting UCHL5 and USP14, plays a crucial role in the anti-inflammatory and anti-thrombotic mechanisms. Treatment with b-AP15 significantly reduces levels of TNF-α and IL-6 by regulating the NF-κB pathway in LPS-stimulated THP-1 and macrophages. b-AP15 improves the survival rate of mice with sepsis induced by high-density LPS (80). Furthermore, b-AP15 inhibits platelet aggregation induced by thrombin, collagen, and ADP, while also accompanied by curtailed procaspase-activating compound-1 binding to activated IIb/IIIa and inhibition of P-selectin translocation to the platelet surface (81).

### 5.2 Emerging therapeutic strategy

The UPS has emerged as a promising target for therapeutic intervention in various diseases, including APS. However, the clinical application of UPS inhibitors, such as bortezomib, is often hampered by the development of drug resistance. The limitations have spurred the adoption of innovative UPS-based technologies aimed at promoting protein degradation or stability. Among these, molecular glue degraders and PROTACs (proteolysis-targeting chimeras) have shown potential in inducing the targeted degradation of proteins, while DUBTACs (deubiquitinase-targeting chimeras) aim to increase protein stability (82).

PROTACs are designed as conjugates of target protein ligands, linked to E3 ligase ligands via a linker. This novel therapeutic strategy induces the degradation or reduction of pathogenic proteins without directly inhibiting their functional activity, offering a new approach to overcome the limitations associated with traditional protein inhibitors (83). As a key upstream regulator of NF-κB activation, BTK (Bruton’s tyrosine kinase) plays a significant role in modulating the inflammatory responses of innate immune cells. Inhibition of BTK has been demonstrated to attenuate the secretion of pro-inflammatory cytokines, establishing it as a viable therapeutic target for the treatment of acute and chronic inflammatory conditions. A research team discovered BTK PROTACs based on ibrutinib that recruit the cereblon ligase, suppressing the secretion of proinflammatory cytokines and reducing inflammatory responses in mouse models (83). HDAC6 (histone deacetylase 6) is implicated in the activation of the NLRP3 inflammasome, indicating that targeting HDAC6 could be beneficial for treating inflammatory diseases. Utilizing a PROTAC strategy, Z. Cao et al. (84) have reported a low cytotoxicity HDAC6 degrading agent by binding a selective HDAC6 inhibitor derived from natural product indigo carmine to the CRBN E3 ligand pomalidomide. The HDAC6 PROTAC can effectively and selectively reduce HDAC6 levels in activated THP-1 cells and diminish NLRP3 inflammasome activation induced by LPS in mice. This provides initial evidence that HDAC6 PROTACs may offer a novel therapeutic strategy for NLRP3 inflammasome-related diseases, potentially serving as an alternative treatment for APS.

Despite the potential of PROTACs, the high molecular weight of PROTACs and their requirement for target protein to have a binding pocket limit their application. Molecular glues are expected to overcome these obstacles. Thalidomide, initially developed in the 1950s for morning sickness and currently used for leprosy and multiple myeloma, is a prime example of a molecular glue. Thalidomide and its analogues can inhibit the synthesis of TNF-α by activated monocytes and have demonstrated efficacy in autoimmune diseases such as RA (85), SLE (86), and systemic sclerosis, although the precise mechanisms were previously unknown (87). In 2010, Japanese scientists revealed that thalidomide could act as a “molecular glue”, interacting with E3 ligase CRBN and IKZF1/3 (ikaros family zinc finger 1/3) proteins, inducing the two proteins to approach each other. Subsequently, CRBN ubiquitinated IKZF1/3, leading to the ubiquitination and subsequent degradation of IKZF1/3 by proteasomes, thereby exerting anti-angiogenic, anti-proliferative, and anti-inflammatory effects (88). Thus, the potential role of molecular glues in treating autoimmune diseases has been continuously explored. Chinese scientists have also established DUBTAC platforms based on the DUBs such as OTUB1 and USP7, significantly broadening the scope of targeting protein stability (89, 90). However, research on DUBTAC technology is still in its early stages, and its application in autoimmune disease treatment requires further validation through laboratory studies and clinical trials. The exploration of UPS-based therapeutic strategies, including PROTACs, molecular glues, and DUBTACs, represents a significant advancement in the treatment of APS and other inflammatory conditions. These innovative approaches offer new avenues for modulating protein stability and interactions, potentially leading to more effective and targeted therapies in the future (5).

## 6 Conclusion

Ubiquitination serves as a pivotal post-translational modification mechanism, exerting its influence by modulating protein stability and activity. It plays a significant role in the pathogenesis of APS through the regulation of thrombotic and inflammatory responses in key cellular components, including monocytes, neutrophils, ECs, and trophoblast cells. E3 ubiquitin ligase is the most abundant and specific molecule in the UPS family, and its research in autoimmune diseases is also the most extensive. In contrast, the investigation of E1 ubiquitin-activating enzymes and E2 ubiquitin-conjugating enzymes in rheumatic diseases remains insufficient, which may be limited by the non-specific effects of E1 and E2 enzymes. Notably, recent studies have identified a link between the ubiquitin-conjugating enzyme UBE2 and the autoimmune skin condition psoriasis vulgaris, marking a significant advance in understanding the role of ubiquitin-conjugating enzymes in autoimmune pathologies (91). It is worth noting that a single enzyme may target multiple proteins, and conversely, a single protein target may be regulated by various UPS components. The integration of UPS inhibitors with traditional anticoagulant and anti-inflammatory therapies may offer a novel strategy for APS management. Moreover, the advent of novel therapeutic technologies based on the UPS degradation system, such as PROTACs, has garnered significant scientific interest. These technologies hold promise for degrading a range of cellular target proteins, demonstrating potential in treating tumors, autoimmune, and inflammatory diseases. Future breakthrough in this field likely lies in the discovery of a more diverse array of E3 ligases and E3 ligands that are characterized by low molecular weight and high activity. This will expand the target and indication spectrum, enhance drug efficacy, and improve safety profiles. A deeper comprehension of the UPS mechanisms and clinical evidence in autoimmune diseases may well catalyze the next revolutionary phase in drug discovery and development.
